# FRA2A Is a CGG Repeat Expansion Associated with Silencing of *AFF3*

**DOI:** 10.1371/journal.pgen.1004242

**Published:** 2014-04-24

**Authors:** Sofie Metsu, Liesbeth Rooms, Jacqueline Rainger, Martin S. Taylor, Hemant Bengani, David I. Wilson, Chandra Sekhar Reddy Chilamakuri, Harris Morrison, Geert Vandeweyer, Edwin Reyniers, Evelyn Douglas, Geoffrey Thompson, Eric Haan, Jozef Gecz, David R. FitzPatrick, R. Frank Kooy

**Affiliations:** 1 Department of Medical Genetics, University of Antwerp, Antwerp, Belgium; 2 Medical and Developmental Genetics Section, MRC Human Genetics Unit, IGMM, University of Edinburgh, Edinburgh, United Kingdom; 3 University of Southampton, Centre for Human Development, Stem Cells and Regeneration, Human Genetics, Southampton, United Kingdom; 4 Department of Tumor Biology, Institute for Cancer Research, Oslo University Hospital, Oslo, Norway; 5 Genetics and Molecular Pathology, SA Pathology, Adelaide, South Australia, Australia; 6 Department of Paediatrics, The University of Adelaide, Adelaide, South Australia, Australia; 7 Department of Paediatrics and Child Health, Flinders University, Adelaide, South Australia, Australia; 8 South Australian Clinical Genetic Service, SA Pathology (at Women's and Children's Hospital), Adelaide, South Australia, Australia; The Hospital for Sick Children and University of Toronto, Canada

## Abstract

Folate-sensitive fragile sites (FSFS) are a rare cytogenetically visible subset of dynamic mutations. Of the eight molecularly characterized FSFS, four are associated with intellectual disability (ID). Cytogenetic expression results from CGG tri-nucleotide-repeat expansion mutation associated with local CpG hypermethylation and transcriptional silencing. The best studied is the FRAXA site in the *FMR1* gene, where large expansions cause fragile X syndrome, the most common inherited ID syndrome. Here we studied three families with FRA2A expression at 2q11 associated with a wide spectrum of neurodevelopmental phenotypes. We identified a polymorphic CGG repeat in a conserved, brain-active alternative promoter of the *AFF3* gene, an autosomal homolog of the X-linked *AFF2*/*FMR2* gene: Expansion of the *AFF2* CGG repeat causes FRAXE ID. We found that FRA2A-expressing individuals have mosaic expansions of the *AFF3* CGG repeat in the range of several hundred repeat units. Moreover, bisulfite sequencing and pyrosequencing both suggest *AFF3* promoter hypermethylation. cSNP-analysis demonstrates monoallelic expression of the *AFF3* gene in FRA2A carriers thus predicting that FRA2A expression results in functional haploinsufficiency for *AFF3* at least in a subset of tissues. By whole-mount in situ hybridization the mouse *AFF3* ortholog shows strong regional expression in the developing brain, somites and limb buds in 9.5–12.5dpc mouse embryos. Our data suggest that there may be an association between FRA2A and a delay in the acquisition of motor and language skills in the families studied here. However, additional cases are required to firmly establish a causal relationship.

## Author Summary

Some human genetic diseases are caused by dynamic mutations, or expansions of a short repeated sequence in the genome that can be unstably passed on from generation to generation. A subset of these dynamic mutations known as fragile sites can be seen as a break or gap on the chromosome when cells are cultured under specific conditions. To date eight folate-sensitive fragile sites (FSFS) have been characterized, and all are due to CGG-repeat expansions within the 5′ UTR or promoter region of the respective gene. When the repeat expands in size, it becomes hypermethylated and the adjacent gene or genes are transcriptionally silenced. For at least four of the eight known fragile sites this silencing of the associated gene(s) lead to intellectual disability syndromes such as fragile X. In this work we describe molecular characterization of an autosomal FSFS called FRA2A on chromosome 2. As the molecular cause of FRA2A, we identify an expansion of a CGG repeat which subsequently results in silencing of the neighbouring gene *AFF3*. This gene is one of the four autosomal paralogss of the *AFF2/FMR2* gene which, when mutated, is the cause of the FRAXE syndrome. We find that FRA2A expression is associated with highly variable developmental anomalies in the three FRA2A families studied.

## Introduction

Dynamic mutations are heritable unstable expansions of short, genomic repeat sequences. Various pathogenic mechanisms have been associated with dynamic mutations [1], [2] and at least 40 neurological, neurodegenerative and neuromuscular disorders are known to be caused by these types of mutations [3], [4]. Expansions of these unstable sequences may occur in promoters, coding regions, introns and 3′ and 5′ untranslated regions (UTR) of genes [5], [6], [7]. Known and putative disease mechanisms include aberrant splicing [8], loss or gain of function of the encoded protein [9], [10], the expanded repeat itself [11] or its RNA transcript [12], [13] and Repeat Associated Non-ATG translation (RAN translation) [14], [15]. The size threshold at which a repeat becomes unstable and/or pathogenic varies widely, from the expansion of only a few trinucleotide repeats in e.g. *ARX*-associated infantile epileptic encephalopathy (MIM 308350) to over a thousand repeats in e.g. *DMPK*-associated myotonic dystrophy (MIM 160900), *FXN*-associated Friedreich ataxia (MIM 229300) and *FMR1*-associated fragile X syndrome (MIM 300624) [16], [17], [18].

Fragile sites represent a specific subset of dynamic mutations that are visible as gaps or breaks on metaphase chromosomes from cells cultured under specific conditions. Fragile sites are categorised by the nature of the inducing culture condition and the population frequency of the mutation [19]. FRAXA is a rare, folate sensitive fragile site (FSFS) associated with a trinucleotide repeat (CGG) expansion mutation in the 5′ UTR of the *FMR1* gene resulting in fragile X syndrome, the most common inherited intellectual disability syndrome [20]. Twenty-six other FSFS have been reported cytogenetically but only eight of these have been molecularly characterized: FRAXA [20], FRAXE [21], FRAXF [22], FRA16A [23], FRA11B [24], FRA10A [25], FRA12A [26] and FRA11A [27]. To date, all characterized FSFS are due to a CCG/CGG trinucleotide repeat expansion. The expanded repeat and any adjacent CpG island become hypermethylated and transcriptionally silenced at a locus-specific repeat size-threshold [28]. At least four of the eight characterized rare, folate sensitive fragile sites are associated with a neurodevelopmental disorder. The relevance of folate sensitive fragile sites to intellectual disability (ID) is strengthened by five independent population studies that have all shown that autosomal folate sensitive fragile sites are overrepresented in people with ID compared to control groups without ID, with a prevalence of 1.2% and 0.27% respectively [reviewed in 29]. It thus seems likely that as yet uncharacterized CGG/CCG repeat expansions may be associated with neurodevelopmental problems.

An autosomal FSFS on chromosomal band 2q11.2-q12 has been previously described [30], [31], [32]. We studied three families with FRA2A-expression and a wide spectrum of neurodevelopmental and other anomalies. We identified expansion of an intronic CGG repeat leading to hypermethylation of at least one promoter of the *AFF3* gene in all FRA2A carriers and we hypothesise that the associated transcriptional silencing of *AFF3* in the brain may be responsible for some of the developmental features observed in FRA2A carriers.

## Results

### FRA2A is due to expansion of a polymorphic CGG repeat within AFF3

**Figure 1:**
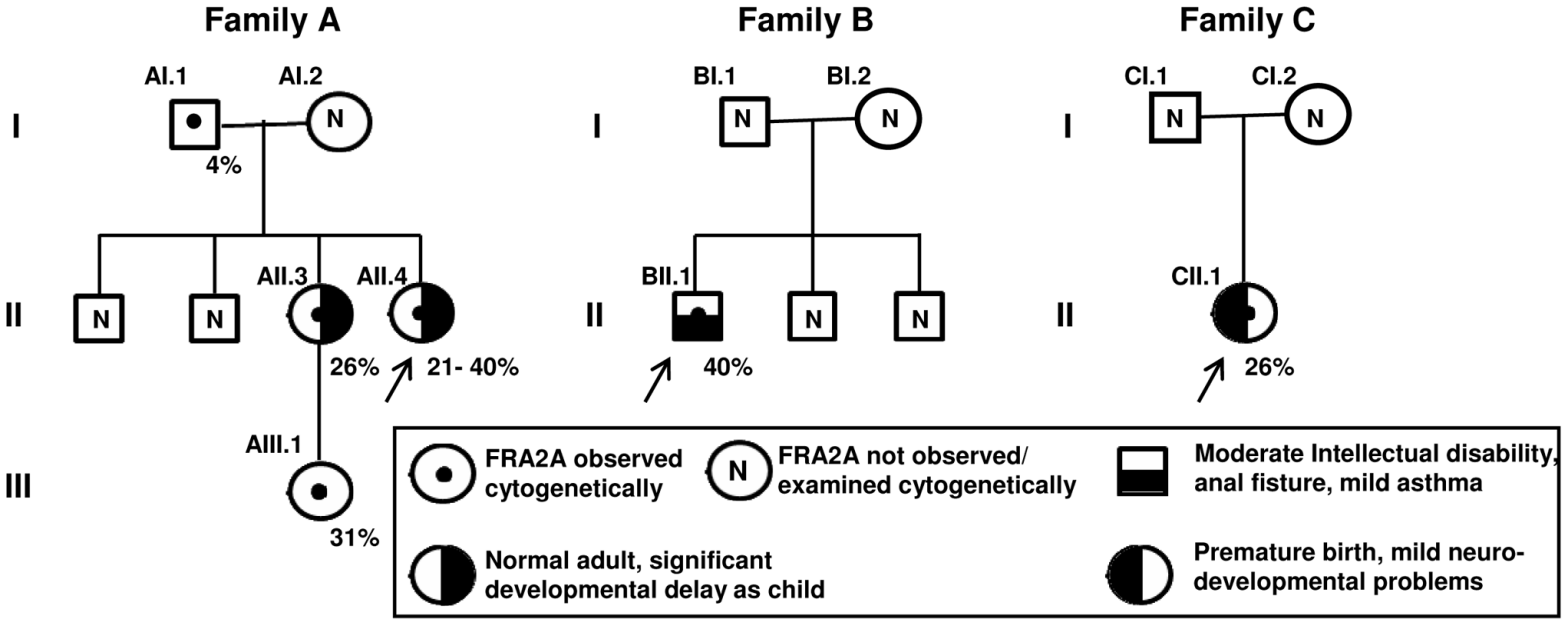
Description of FRA2A Families A–C. Females are represented by *circles*, males by *squares*. The percentage of cells showing FRA2A expression are indicated on the bottom right-hand side of the symbol. As discussed in the text the 4% FRA2A expression seen in individual AI.1 represents a false positive. The case number used to indicate the FRA2A carriers in the text is given on the top left-hand side of the symbol. N within a symbol indicated individuals expression of the fragile site was not examined or observed. The associated phenotypes in individuals AII.3, AII.4, BII.1 and CII.1 are detailed in the boxed symbol key. The proband in each family is arrowed.

**Figure 2:**
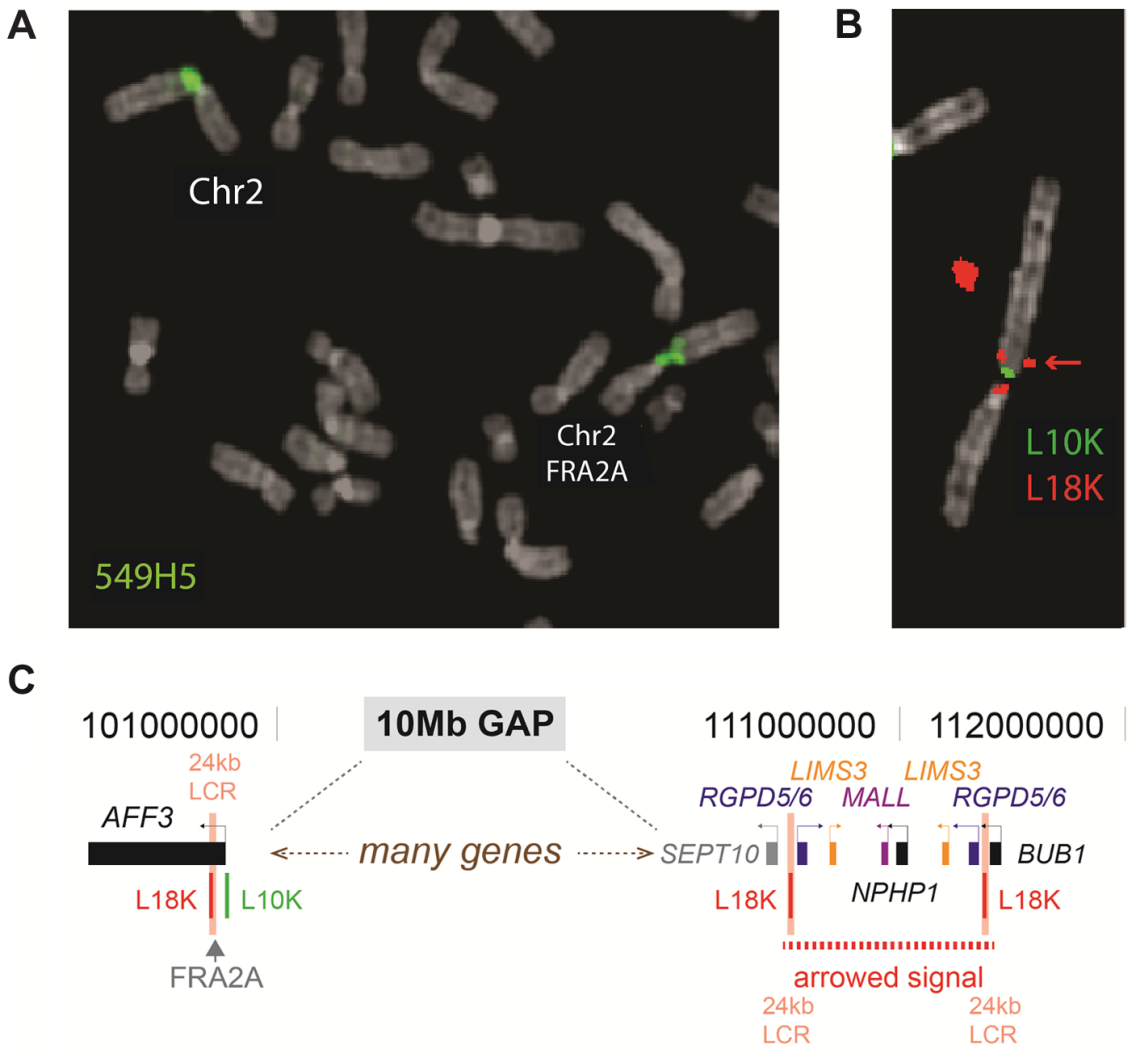
FISH analysis of the FRA2A fragile site. **A.** The BAC-clone 549H5 (labelled green) spans the fragile site FRA2A. **B.** FISH analysis using the 10 kb L10K (labelled green; chr2: 100721983–100733233; hg19) and 18 kb L18K (labelled red; chr2: 100700447–100718834; hg19) PCR-generated probes, targeted to map either side of FRA2A. The additional telomeric FISH signal on the red channel (red arrow chr2: 110520380–110538822 and chr2:111347822–111366260; hg19) is the result of a 24 kb low copy repeat (24 kb LCR pink text) encompassing L18K. **C.** A schematic representation of the position of the LCR.

Using the simple repeat track on UCSC genome browser (GRCh37/hg19) we identified three candidate CGG repeats in the FRA2A containing region (2q11-12). One of these repeats is located within the *LAF4/AFF3* gene (chr2:100721261–100721286; hg19), an autosomal homolog of the FRAXE-associated *FMR2/AFF2* gene. In order to determine whether this CGG-repeat is expanded in FRA2A we used metaphase FISH analysis on a FRA2A-expressing individual (Figure 1; AII.3) with the BAC clone RP11-549H5 (chr2:100,588,792–100,759,365; hg19) encompassing the repeat. The FISH signal spanned the FRA2A fragile site (Figure 2A). Consistent with this the FISH signals from probes RP11-436F6 (AC010736) and RP11-506F3 (AC074387) were centromeric and telomeric to FRA2A, respectively. To establish co-location of the CGG repeat with the fragile site, long-range PCR-generated probes L10K (chr2:100721983–100733233; hg19) and L18K (chr2:100700447–100718834; hg19) were targeted to the genomic regions on either side of the (CGG)_n_ repeat. These probes did indeed flank the fragile site, locating FRA2A to a 3.1 kb interval within the *AFF3* gene (Figure 2B). The second red (L18K) FISH signal observed at 2q13 (Figure 2B) is the result of two copies of a 24 kb low copy repeat flanking the *NPHP1* gene (Figure 2C) (chr2: 110520380–110538822 and chr2:111347822–111366260; hg19). A copy of this sequence is also located adjacent to the CGG-repeat within the *AFF3* gene in the region covered by L18K.

**Figure 3:**
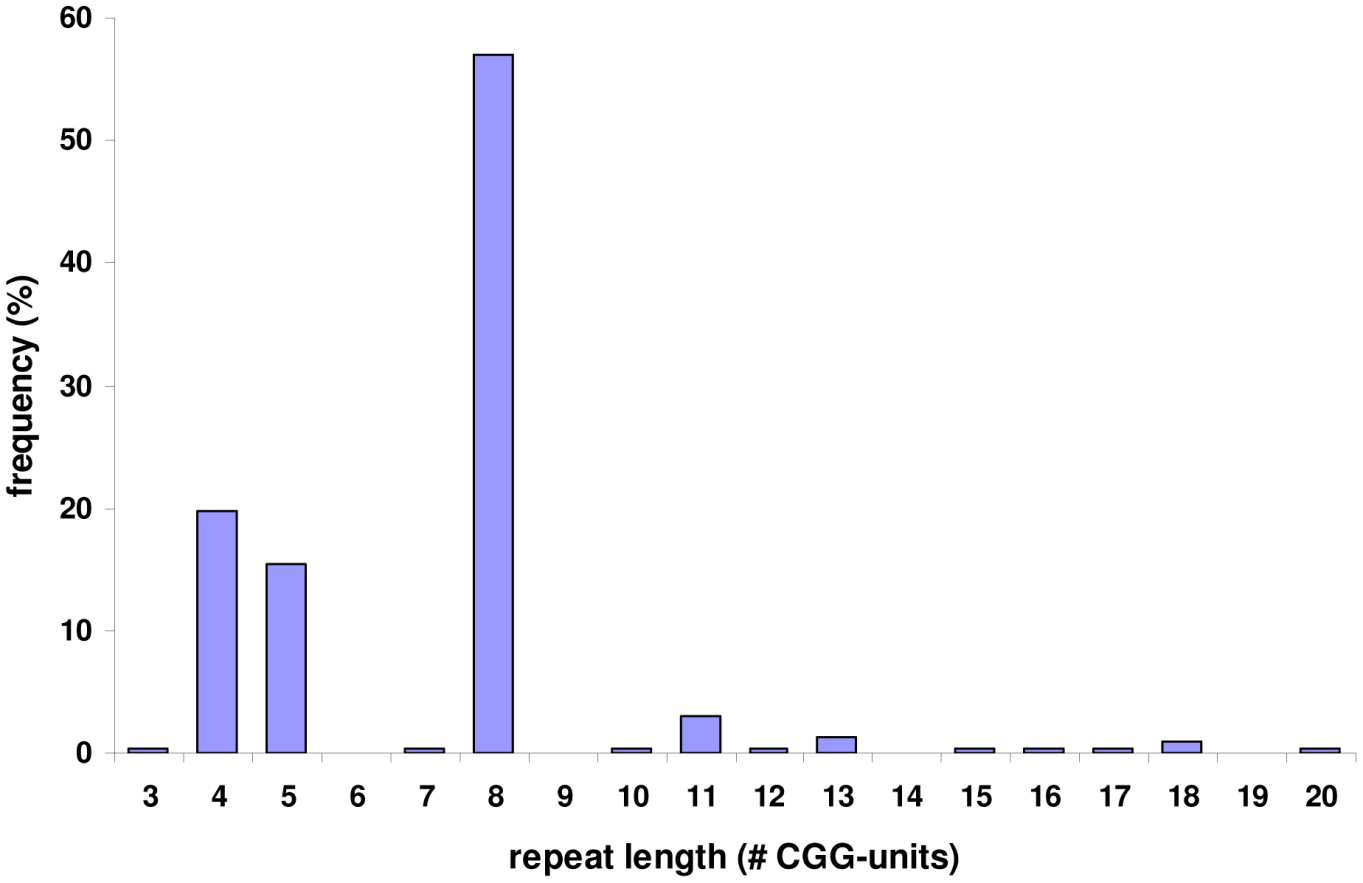
Allele frequencies of the FRA2A associated CGG repeat in a population of 100 control individuals. PCR amplification of the repeat and subsequent sequencing in 200 control chromosomes revealed that it is highly polymorphic with a length ranging from 3 to 20 copies. The most commonly found allele contains eight repeated units.

PCR-based amplification and sequencing of the *AFF3* CGG repeat in 200 control chromosomes revealed it to be highly polymorphic with a length ranging from 3 to 20 copies (Figure 3). The most frequent CGG allele contains eight repeats (as does the genomic reference sequence; chr2:100721261–100721286; hg19). To exclude the possibility that apparently homozygous control individuals are in fact heterozygous for the detected allele in combination with a large expansion that is not detected by this protocol, we amplified these samples with gene specific repeat primed PCR (Asuragen Inc., Austin Texas, USA). This protocol enables us to detect expansions up to 300–500 repeats. However, no expanded repeats were detected in control chromosomes, and the genotype distribution agreed with Hardy-Weinberg equilibrium (P>0.05).

**Figure 4:**
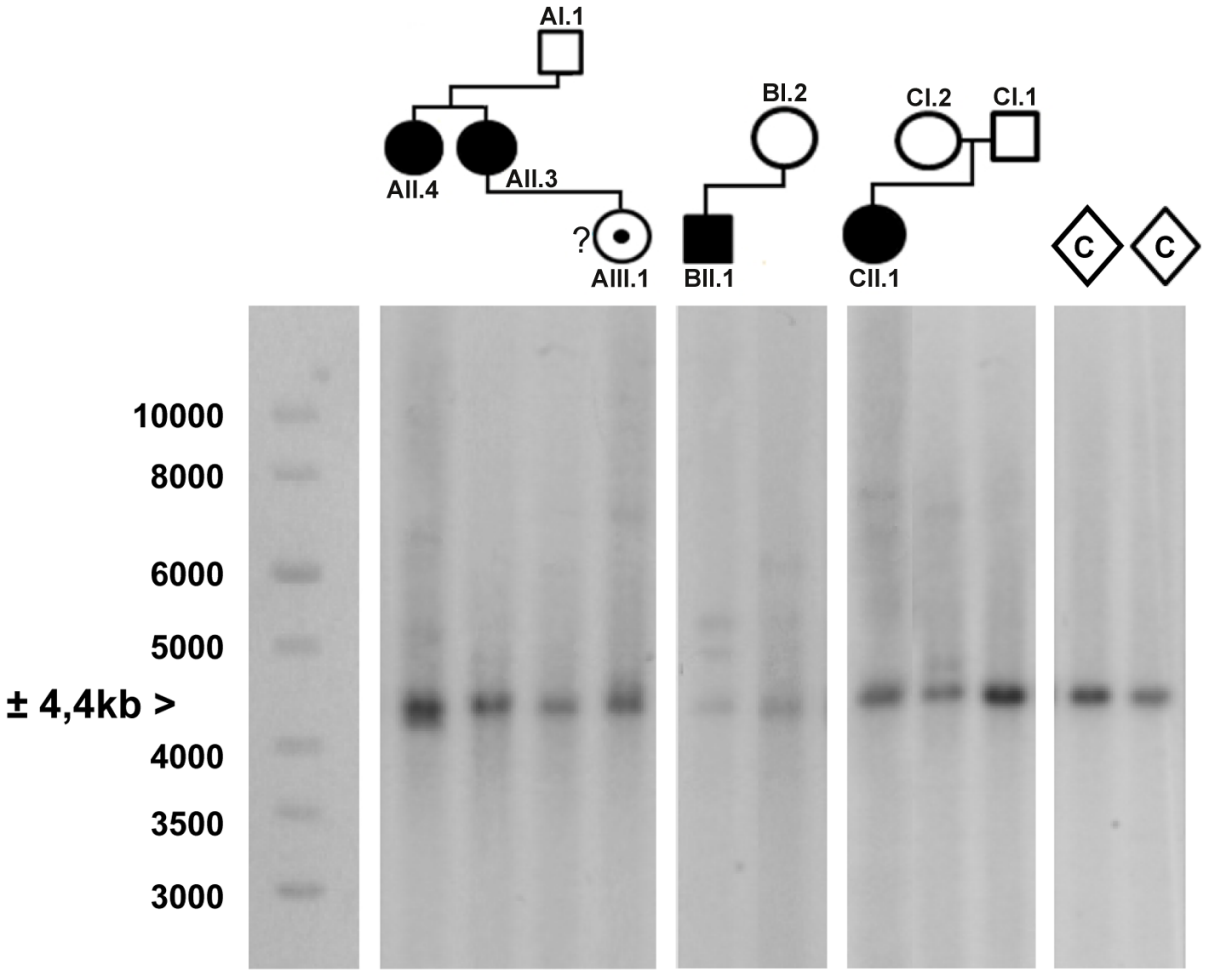
Southern blot analysis of the *AFF3* CGG repeat in all available members of the three families and two unrelated control individuals (C). DNA restriction fragments obtained from blood samples of all available members of family A (lanes 1–4), B (lanes 5–6) and C (lanes 7–9) where blotted with a specific 32P-labeled probe (chr2:100088460–100089451; hg19) after digestion with *Hind*III. Lanes 10 and 11 contain DNA restriction fragments from two unrelated control samples (represented as diamonds) after digestion with *Hind*III. In addition to a normal size allele fragment of 4.4 kb, individuals CII.1, CI.2, BII.1, BI.2, AII.4, AII.3 and AIII.1 show additional fragments of a larger size, indicating repeat expansion. These expanded fragments were not present in the controls and individuals CI.1 and AI.1. A 1 kb length marker is presented at the left of the figure.

PCR amplification of the repeat in the FRA2A-expressing individuals from AI.1, AII.3, AII.4, AIII.1, BII.1 and CII.1 (Figure 1) showed a single CGG allele in the normal size range. An additional smaller fragment was detected in subject AII.4. Sequence analysis of the smaller PCR product showed a 134-bp deletion encompassing the entire CGG repeat as well as some flanking sequences (Figure S1). This deletion was not detected in 800 control chromosomes. To visualize the repeat expansion in the FRA2A-positive individuals, we performed Southern blotting on *Hind*III digested genomic DNA of all available members of the three families (AI.1, AII.3, AII.4, AIII.1, BI.2, BII.1, CI.1, CI.2 and CII.1) and control samples. A 4.4 kb fragment was observed in all cases and controls. In five affected FRA2A-positive individuals we detected additional large fragments or smears compatible with the presence of an expanded allele (Figure 4). Two FRA2A-negative parents of FRA2A-positive individuals (BI.2 and CI.2) showed additional larger fragments indicative of repeat expansion. No evidence of an expanded fragment was observed in the control samples or in FRA2A-negative individual CI.1. Interestingly, individual AI.1 who had been reported as showing a low level of FRA2A-positive cells showed no fragments suggestive of an expansion mutation. A Southern blot of the same samples after *Nco*I digestion gave very similar results (data not shown).

A gene specific repeat-primed PCR assay was used for accurate sizing of the repeat expansion mutations. The mothers in family B and C both showed a slightly expanded allele (±120 and 106 repeat units, respectively) in addition to an allele in the normal size range (15 and 17 repeat units, respectively) while their offspring show one allele of 8 units compatible with paternal inheritance and one allele with a large expansion of more than 300 units (Figure S2). This strongly suggests that the expanded allele in both families was inherited from the mother (Table 1). In family A, the apparently FRA2A-positive individual AI.1 showed no evidence of an expanded allele on either Southern blot analysis or on repeat primed PCR. This apparent discrepancy could be resolved genetically using the microsatellite markers (D2S2209/AFMA246XE9 and D2S2311/AFMB355ZG1, Figure S3). The FRA2A-positive daughters of AI.1, AII.3 and AII.4, were shown to have inherited a different non-expanded allele (8 CGG units) from him, while they share a common allele with the mother (AI.2). Their FRA2A-positive grand-daughter, AIII.1 also inherited this maternal allele. The expansion was therefore probably inherited from AI.2, with the 4% FRA2A expression in AI.1 representing a false-positive cytogenetic result. Unfortunately DNA was not available from AI.2 to determine if she also carried an intermediately expanded allele.

**Table 1:** Sizing the repeat in all available family members with gene specific repeat primed PCR.

	Family A				Family B		Family C		
	I.1	II.3	II.4*	III.1	I.2	II.1	I.1	I.2	II.1
**allele 1**	8	8	8	18	15	8	5	17	8
**allele 2**	8	>300	>300	>300	±120	>300	8	106	>300

Alleles were sized by gene specific repeat primed PCR. As expansions containing over 300 repeated units cannot be reliably sized using this technique, we indicated these alleles as “>300”. Subject A.II.4 is marked with an asterisk as she is mosaic for a 134-bp deletion taking away the entire CGG repeat in combination with a largely expanded allele, and this in addition to a normal sized allele.

### Characterization of the two major AFF3 transcriptional start sites (TSS)

The RefSeq *AFF3* gene model consists of 23 coding exons and two 5′ non-coding exons together spanning 558 kb genomic DNA [33]. Here, the 5′ non-coding exons are named exons 1 and 2 with the first coding exon called exon 3. An AFF3-specific cDNA probe encompassing the final 5 exons was used for northern blot analysis. A major transcript of approximately 8 kb, corresponding to the predicted size was detected in several tissues, including brain, placenta and lung (data not shown).

**Figure 5:**
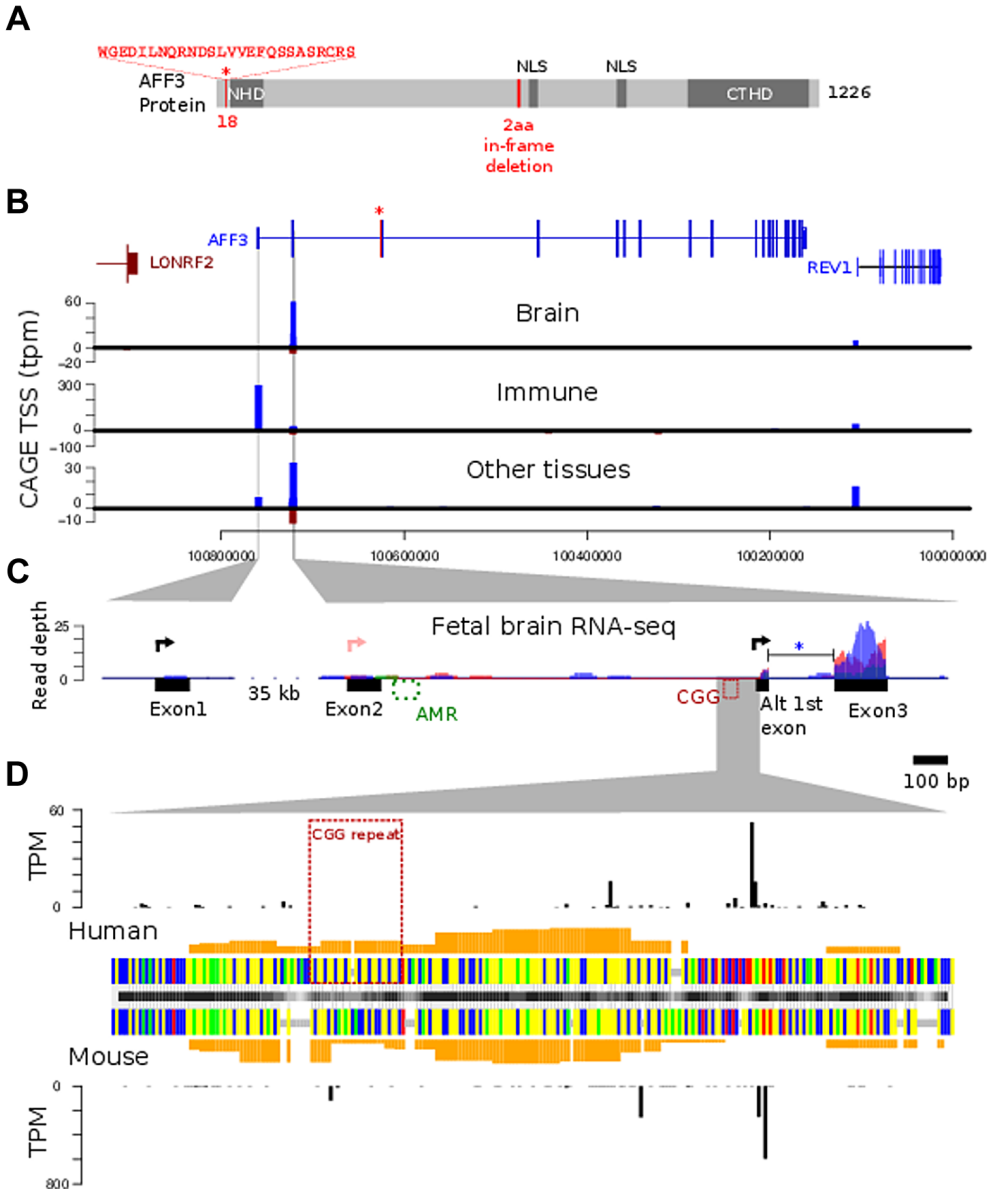
AFF3 protein-domain and gene structure. **A.** diagrammatic representation of the *AFF3* protein, showing the N-terminal homology domain (NHD), C-terminal homology domain (CTHD) and two predicted nuclear localisation signals. The 18 amino acids encoded by the alternatively spliced exon 4 are represented as an “insertion” at position 18 of isoform 1. The 6-bp in-frame deletion we identified in exon 14 of subjects AI.1 and AII.4, removing two amino acids (position 619 and 621 respectively) is indicated in red. This deletion was predicted to be benign. **B.** Genomic structure of *AFF3* with the alternately used spliced exon 4 shown in red with asterisk. AFF3 exons 2, 3 and the alternate first exon are not separately resolvable at this resolution but shown in detail in panel C. Transcription left to right is shown in blue, right to left in brown. CAGE tag defined transcription start sites are shown aligned with annotated gene structure (blue forward strand transcription, brown reverse strand transcription shown with negative counts). Y-axis values show average tags per million (TPM) from CAGE libraries from the indicated tissue groups. **C.** Finer details of the *AFF3* TSS regions are shown along with histograms of human fetal brain RNA-seq read coverage; three replicates are coloured separately. Splicing of the intron between the alternate first exon and exon 3 (blue asterisk) was supported by 9 independent RNA-seq reads and found in all three replicates. The CGG repeat and abnormally methylated region (AMR) are shown in red and green respectively, The major transcription start sites are shown as black arrowed lines. There is no supporting evidence for the RefSeq TSS represented by the pink arrowed line in our data. **D.** Alignment of human CGG repeat region and associated TSS with the orthologous mouse region. Nucleotides are color coded (A = green, G = yellow, C = blue, T = red, alignment gaps are grey). Orange histograms show the predicted G-quadruplex forming potential of the human and mouse sequences. Outer histograms show CAGE tag 5′ ends at single nucleotide resolution in both human (top) and mouse (bottom). TPM counts shown are the average from brain derived CAGE libraries in each species and represent the precise location of transcription initiations.

To determine the precise location of the *AFF3* transcriptional start sites (TSSs) in relation to the expanded repeat we used Cap Analysis of Gene Expression (CAGE) data from the FANTOM4 consortium. FANTOM4 produces sequence tags from the 5′ end of mRNAs from many different tissue sources and species and maps these to the reference genome [34]. Mapped CAGE tags reveal the sites of transcription initiation at single nucleotide resolution and provides a semi-quantitative measure of steady state mRNA levels for those transcripts using a tags per million (TPM) metric. The TPM scores for three different human tissue groups (brain, immune and other tissues) are plotted in Figure 5B. *AFF3* is transcribed in telomeric to centromeric orientation and the x axis of these graphs represent the hg19 genomic coordinates. The location of the 25 annotated exons of the RefSeq model of the AFF3 gene (NM_001025108) is represented above the graphs using the same genomic coordinates. To assess the transcriptional activity of *AFF3* during early brain development we mapped transcriptome sequencing reads of mRNA (RNA-seq) from three different human fetal brain samples to the regions surrounding the TSS identified by CAGE (Figure 5C).

CAGE tag sequencing demonstrates two robust TSSs. The most 5′ TSS corresponds to the 5′ end of exon 1 at position chr2:100759169 (GRCh37/hg19). This TSS is highly expressed in immune tissues with a mean of 300 tags per million shown by the blue bar in the middle graph (Figure 5B). There is no obvious transcription of exon 1 on RNA-seq from human fetal brain (Figure 5C, left-hand side). A second robust TSS was identified in CAGE data from brain and other tissues which mapped within intron 2 as shown by the blue bars in the top and bottom graphs in Figure 5B. The highest levels are seen in the brain (mean of 60 tags per million). An expanded representation of this region is shown in the right-hand side of Figure 5C. This shows no evidence of transcription of exon 2 but strong expression in exon 3. This also shows evidence of an alternative exon 1 immediately 3′ to the TSS (Figure 5C, right hand side, black arrow). The TSS lies immediately downstream of the CGG repeat (Figure 5D) suggesting this expansion prone repeat is located within the core of an alternate *AFF3* promoter.

### The location and tissue specific activity of the *AFF3* promoters are similar in mouse *Aff3*

FANTOM4 CAGE data also encompasses a range of mouse tissues. From this we can demonstrate that both the exon 1 TSS and intron 2 TSS are evolutionarily conserved and functional TSSs. Although the CGG repeat itself is not conserved, a region of low compositional complexity flanked by highly constrained non-coding sequences is a conserved feature of the intron 2 TSS promoter (Figure 5).

**Figure 6:**
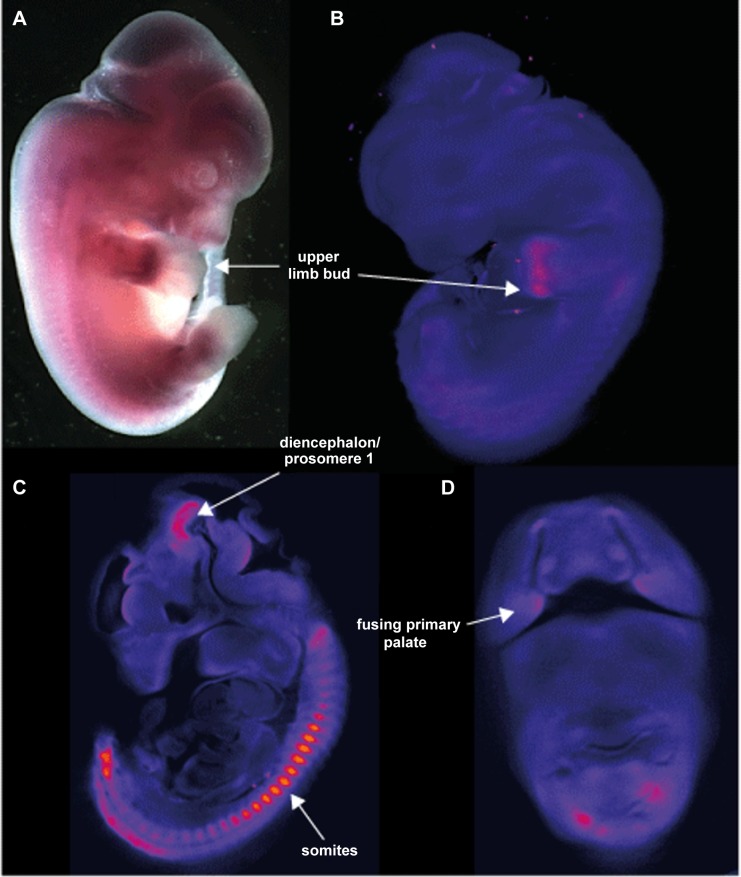
Site and stage specific expression of *Aff3* in mouse embryos. **A.** photomicrograph of a lateral view of a 12.5*Aff3*. The only area of strong expression at this stage is in the hand plate. **B.** show a false coloured but unthresholded image of the brightfield optical projection tomography (OPT) scan of a 11.5 dpc embryo with digital sagittal and coronal sections from the same embryo shown in **C** and **D.** Strong expression is seen in the somites, upper limb bud, the fusing primary palate and the diencephalon and prosomere1 regions of the developing brain.

Whole mount *in situ* hybridization (WISH) using riboprobes targeted to the 3′UTR of *Aff3* was used to determine the developmental expression pattern in mouse embryos at 9.5, 10.5, 11.5 and 12.5 days post coitus (dpc). This has shown site and stage specific expression of *Aff3*. The most striking areas of expression are in the somites, the upper limb buds, the diencephalon/prosomere I and the fusing primary palate (Figure 6).

### AFF3 promoter regions are hypermethylated in individuals with the FRA2A CGG-repeat expansion

In all rare, folate-sensitive sites characterised to date, CGG repeat expansions are associated with hypermethylation of the surrounding CpG island. Bisulfite sequencing indicated hypermethylation of the CpG island in all five affected FRA2A carriers AII.3, AII.4, AIII.1, BII.1 and CII.1, while in healthy control individuals this region was not methylated (figure S4). In order to quantify the methylation level, we subsequently subjected all samples to pyrosequencing. This technique allows accurate quantification of methylation across individual CpG sites [35]. A methylation frequency of 50% would be consistent with complete methylation of the expanded allele as all affected individuals in this study are heterozygous. We analyzed a short region of genomic DNA (chr2:100721843–100721885; hg19) adjacent to the CGG repeat in all available family members, containing four analysable CpG dinucleotides (Figure S1 and Table 2). Methylation percentages are congruous across the 4 CpG-sites in each individual (p-values ranging from 0.417 to 1.000) and are consistent with hypermethylation of the CGG repeat region in individuals carrying an expanded allele. There is some suggestion that the methylation frequency may be increasing upon generational transmission.

**Table 2:** Pyrosequencing reliably quantified the methylation of four consecutive CpG dinucleotides per individual (chr2:100721843–100721885; hg19).

	**CG**CAA**CG**GAGAGCAGGTC**CG**GGTGGAAGAGGTTTCCTCCG**CG**C				
**Family A**					
I.1	5±1,6	4±1,3	5±0,5	–	2±0,5
II.3	18±2,0	17±3,8	20±3,4	–	14±2,6
II.4	24±2,9	21±2,9	24±3,8	–	18±2,5
III.1	26±2,7	22±1,5	25±3,0	–	21±1,1
**Family B**					
I.2	27±3,1	26±3,1	28±3,6	–	22±3,1
II.1	45±0,4	44±3,2	48±2,4	–	33±2,4
**Family C**					
I.1	5±1,7	3±1,3	3±1,7	–	2±2,8
I.2	40±3,6	40±4,4	34±4,3	–	35±3,6
II.1	54±4,5	52±2,8	60±3,5	–	45±3,4

A fifth CpG site is preceded by a 7 base pair T stretch (after bisulfite conversion), making it impossible to assess the exact percentage of cytosines compared to thymines for that particular site due to the limitations of the pyrosequencing technique. For each individual at least three separate analysis of different bisulfite conversions were performed. Average methylation percentages and confidence intervals are presented in this table. Methylation cut-off value was set at 10% according to the manufacturers guidelines.

To exclude a non-specific effect of increasing age on the methylation of this region, we pyrosequenced 72 individuals from 24 unrelated two-generation families. The ages within this control group varied between 0 and 11 years for the children and between 23 and 53 years for the parents, which is comparable to the age distribution in our FRA2A families at the time of DNA collection. No methylation above the threshold was detected in any control individual (data not shown). In the FRA2A-carriers the methylation level for each of the four CpG sites differed significantly from the level determined in this control population (p-values≤0.001 for CpG site 1,2 and 4 and p<0.004 for CpG site 3), suggesting that expansion of the repeat is associated with hypermethylation of the region surrounding the repeat.

In AII.4, mosaic for a CGG-repeat expansion and a deletion, the promoter region on the expanded allele was hypermethylated as determined by bisulfite sequencing. The allele with the 134-bp deletion was not methylated, as determined by Southern blotting after double digestion with *BamH*I and *Not*I (data not shown).

### Monoallelic expression of the AFF3 gene in FRA2A carriers

**Figure 7:**
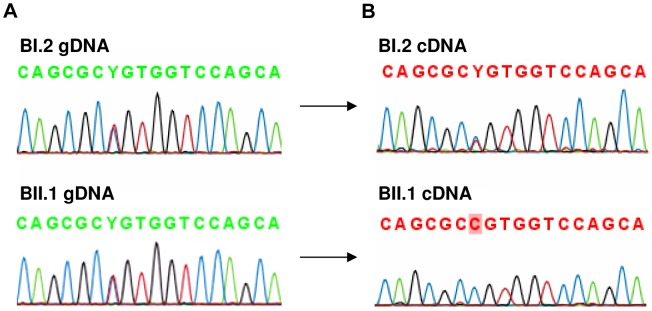
Analysis of rs4851214 which maps to coding exon 14 of *AFF3* using paired genomic DNA and cDNA templates from the unaffected carrier mother BI.2 and the affected carrier son BII.1 from family B. **A:** Both BI.2 and BII.1 are heterozygous for SNP rs4851214 in genomic DNA as T and C peaks are visible at this site. **B:** Analysis of cDNA from subject BII.1, shows that only the C allele could be detected in this patient, indicating monoallelic expression, while in cDNA of subject BI.2 the heterozygous T/C signal was observed, indicating transcription of both alleles.

The level of *AFF3* expression in lymphoblastoid cell lines is too low to be reliably measured by RT-qPCR. To determine if FRA2A results in transcriptional silencing of *AFF3* in *cis*, we utilized single nucleotide polymorphisms (SNP) mapping within the open reading frame. Two such SNPs were found to be heterozygous in the genomic DNA of affected FRA2A carrier BII.1. Rs4851214 maps to exon 14 and heterozygous individuals display both an T and a C peak (c.1499T/C) on Sanger sequencing, while rs13427251 maps to exon 25 and heterozygotes for this SNP show both an A and a T peak (c.5475A/T). Sequencing cDNA from BII.1, revealed only a C peak at rs4851214 (Figure 7) and only the A allele peak was seen for rs13427251. These results are consistent with monoallelic expression of *AFF3* in this individual. Genomic DNA from BII.1's mother (BI.2) is homozygous for the rs13427251 T allele (g.5475T/T) indicating that it is the maternal T allele carrying the CGG expansion that is silenced in BII.1. cDNA of BI.2 showed a heterozygous signal for rs4851214 (c.1499T/C) indicating that both *AFF3* alleles are transcribed in the mother despite partial methylation of her expanded allele.

### Analysis of clinical phenotypes associated with FRA2A

Six FRA2A carriers were initially included in this study (Figure 1, Table 3), four in Family A and one each in Families B and C. Individual AIII.1 is currently too young to make any conclusion about cognitive development. Individual A1.1 has no discernible affected phenotype and he also has the lowest expression of the fragile site. The molecular analysis presented above strongly suggests that the 4% apparent expression of this case represents a false positive and so was excluded from the clinical analysis. Two FRA2A carriers AII.3 and AII.4 are adults; both had global delay in their neurocognitive development to a level that merited genetic investigations during childhood and their long-term placement in special educational facilities. However, as adults both of these individuals are functioning at a normal level and are in full time employment. This raises the possibility that they had a true delay in development rather than a fixed disability. Something similar is observed in FRAXE patients as most adult FRAXE males adapt to live a normal life. Individual CII.1 was born prematurely and had significant respiratory distress, which confounds the unambiguous interpretation of the cause of her mild developmental delay. BII.1 has the most significant delay, currently without a plausible non-genetic explanation. Thus all four of the characterized true FRA2A carriers did have significant delay in their motor and language development.

**Table 3:** Clinical findings in FRA2A carriers.

Family	A	A	A	B	C
Individual	II.3	II.4	III.1	II.1	II.1
Sex	Female	Female	Female	Male	Female
Age at last assessment (yrs)	33	31	0,2	12	13
Growth	Normal	Normal	Normal	Normal	Normal
Delayed motor development	+	+	?	+	+
Delayed speech and language development	+	+	?	+	+
Intellectual disability	−	−	?	Moderate	Mild
Required special educational support	+	+	?	+	−
General health	Good	Good	Good	Reasonable	Reasonable
Other features				anal fissure	macrocephaly, prematurity, hyperreflexia
FRA2A expression in peripheral blood lymphocytes	26%	40%	31%	40%	26%

To determine whether the FRA2A carriers with neurodevelopmental anomalies had additional mutations in the protein coding region of the *AFF3* gene, mutation analysis of all coding exons was performed. No sequence abnormalities were detected in any of the affected FRA2A carriers, except in subject AII.4, in which a 6-bp deletion was identified in exon 14, removing two amino acids: Threonine and Alanine (position 619 and 621 respectively) (Figure 5A). Both amino acids are found in a region, enriched with proline, serine and glutamic acid residues and located between the transactivation domain and the nuclear localisation signal (NLS). According to different prediction software (mutationtaster, mutation assessor, Indelz), the deletion is benign. Moreover, this 6-bp in-frame deletion was also present in the unaffected father (AI.1).

## Discussion

We provide compelling evidence that the molecular basis of the FSFS FRA2A is a CGG repeat expansion in an alternative promoter which is active in the brain and is located in the intron immediately 5′ to the first coding exon of the major *AFF3* transcript. The FRA2A-associated repeat is polymorphic in the general population. Repeat primed PCR showed all individuals with an expansion of over 300 repeat units expressed FRA2A in more than 20% of their cells. The expansion was associated with hypermethylation of a CpG island adjacent to the alternative promoter and, in at least one case, resulted in transcriptional silencing of *AFF3*. These results are consistent with the epigenetic effects that have been described in other FSFS. Within each of the three families studied here higher levels of methylation correlate well with neurodevelopmental delay, higher repeat size and silencing of *AFF3*. However, there are striking disparities in the absolute levels of methylation observed between the families. For example individuals AII.3 and AII.4 both have >300 repeats and had evidence of neurodevelopmental delay during childhood but have lower levels of methylation than BI.2 (∼120 repeats, biallelic expression of *AFF3*) and C1.2 (106 repeats) neither of whom showed evidence of developmental delay. A likely explanation for this is that the assay used here was performed on peripheral blood-derived cells whereas the phenotype in which we are interested is developmental and neural. Many developmental loci appear to show tissue specific differences in DNA methylation [36]. In this regard the ability to model brain development using cerebral organoids from patient-derived pluripotent cells [37] may enable more interpretable transcriptional and epigenomic analyses of the consequences of CGG-repeat expansion on *AFF3*.

Nonetheless, all individuals for whom a significant methylation frequency was measured, show an expanded allele in the pre- or full mutation range. Repeat sizes of >300 do correlate with neurodevelopmental effects and expression of the fragile site in a significant percentage of cells. Carriers of an expanded allele in the premutation range are phenotypically normal but may show lower levels of expression of the fragile site.

In one individual (AII.4) a mosaic deletion of 134-bp removed the entire CGG repeat and the CpG island on the deleted allele remains unmethylated (data not shown). A similar combination of a full mutation with an expanded repeat and a deletion encompassing the repeat has been reported in several fragile X syndrome patients and recently also in a myotonic dystrophy type 1 case [38], [39]. In the fragile X syndrome, the phenotype of deletion cases is often indistinguishable from that of carriers of an expanded repeat, a reported exception being an unaffected individual where the deletion is the major allele present, and the transcription and translation start sites are preserved [40].

*AFF3* belongs to a family of nuclear transcription activating factors including *AFF1/AF4*, *AFF2/FMR2* and *AFF4/AF5q31* [33], [41], [42]. These proteins form super elongating complexes (SEC) with active P-TEFb (positive transcription elongation factor) and AF9/ENL. SECs regulate the induction and expression of different subsets of genes. *AFF3* is the closest paralog of *AFF2*, and is 36% identical on the amino acid sequence level. They share functional domains including the N-terminal Homology Domain (NHD), the C-terminal Homology Domain (CTHD) involved in intranuclear localization and binding of G-quadruplex RNA structures, and the ALF domain that promotes protein degradation through the proteasome pathway and the transactivation domain (TAD). Intriguingly, the highly conserved intron 2 TSS sequences and to a lesser extent the CGG repeat itself, are predicted to have a strong propensity to form G-quadruplex structures (Figure 5D, orange bars) with the most downstream of these being present in the 5′ UTR of the produced transcript. Given that *AFF3* is known to bind G-quadruplexes, there is the potential for *AFF3* autoregulation at this promoter. Both *AFF2* and *AFF3* localize to nuclear speckles and modulate splicing efficiency [43]. The expression pattern of murine *Aff3* overlaps to a considerable extent with that of murine *Aff2* [43], [44].

FRAXE is associated with loss of expression of *AFF2* through dynamic repeat expansion of a CGG repeat in the 5′ UTR. FRAXE causes an X-linked non-syndromic intellectual disability [45]. *AFF2* may play an important role in learning, memory, and language-learning processes [46]. Moreover, rare missense variations in the highly evolutionary conserved sites of the *AFF2* gene might be associated with autism spectrum disorder [47]. The *Aff2* knockout mouse model shows specific cognitive deficits, including an impaired conditioned fear memory over longer periods and enhanced long-term potentiation in the hippocampus [48], [49]. *Aff3* expression is upregulated in cortical neurons during the initial steps of cortical differentiation and is downregulated in postnatal cortex, indicating its involvement in brain development [44]. We have shown that *Aff3* shows strong regional expression in the developing mouse brain.

*AFF3* is thus a reasonable candidate for the neurodevelopmental features seen in FRA2A carriers in our families. Our data predict that FRA2A carriers are haploinsufficient for *AFF3*, at least in a subset of tissues. A confident assignment of causality to the association of *AFF3*-associated repeat expansion mutations with neurodevelopmental anomalies is confounded by the rarity of the fragile site and the strong bias in clinical ascertainment. It is, however, intriguing that delay in motor and language development appears to be a common feature in the individuals presented here and this may represent a true delay in development rather than a fixed disability. *AFF3* deficiency may then be involved in the speed of skill acquisition without impairing the developmental capacity.

A *de novo* microdeletion of 500 kb on chromosome 2q11.2 removing only *AFF3* [50] has been reported in a girl with a severe multisystem disorder consisting of a mesomelic skeletal dysplasia (fibular agenesis, abnormal and triangular tibiae, short neck), urogenital tract malformations, delayed psychomotor development and recurrent apnoea leading to respiratory arrest at the age of 4 months. This clinical presentation is clearly very different to those associated with FRA2A but would be consistent with the expression pattern we demonstrate in mouse embryos. The clinical differences may be explained by the fact that the methylation of the repeat and thus the inactivation of the *AFF3* gene presumably takes place several weeks after fertilization, so that development during the first weeks is not affected [51]. It is also plausible that the transcriptional silencing associated with FRA2A may by tissue specific given that the alternative promoter that is immediately adjacent to the expansion mutation shows evolutionarily-conserved tissue-specific activity, and appears to be the main driver of *AFF3/Aff3* transcription in the brain in humans and mouse.

Both rare and common fragile sites often co-localize with evolutionary breakpoints as was postulated previously by Ruiz-Herrera et al. [52], [53]. We have shown through FISH and BLAST-analysis that the region close to the *AFF3* repeat is indeed involved in a chromosomal rearrangement including a duplication and inversion of a 24 kb sequence from 2q13 to 2q11.2 followed by an ancestral head-to–head chromosomal fusion that led at 2q13 that led to the formation of human chromosome 2. The 2q11.2 breakpoint of this rearrangement falls within base pairs of the repeat and is also present in other primates. The 2q13 region also co-localizes with FRA2B, an as yet to be characterized rare fragile site of the same type.

In conclusion, we report a CGG repeat expansion mutation as the molecular cause of the fragile site FRA2A. FRA2A expression is associated with methylation of an *AFF3* promoter and apparent transcriptional silencing of *AFF3*. It is currently difficult to unequivocally link FRA2A to a specific neurodevelopmental phenotype but it is plausible that haploinsufficiency for *AFF3* in the developing brain is related to a true developmental delay and possibly mild intellectual disability.

## Materials and Methods

### Ethics statement

The ethics committees of the participating study centers approved the study protocol and all participants gave their written informed consent. The study was in accordance with the principles of the current version of the Declaration of Helsinki. The fetal brain tissue was collected with informed written consent and ethical approval by Southampton and South West Hants LREC. The fetal tissue was obtained following surgical termination of pregnancy and staged according to the Carnegie Classification [54], [55].

### Family description: Clinical diagnosis and chromosome analysis

#### Family A

The proband (AII.4, Figure 1) was originally investigated at the age of 7.5-years for mild to moderate learning disability and enuresis. She was born at term following an uneventful pregnancy. There were no neonatal problems but her early motor and language development was reported to be slow. Her general health was good and her weight was on the 25^th^ centile for her age. She attended a school for children with special educational needs. When last seen at the age of 20-years she was healthy and was working as a checkout operative in a high-street store and had no evidence of a significant functional neurocognitive deficit. Her elder sister (AII.3) had been investigated several years earlier for moderate global learning disability and had attended the same a school for children with special educational needs. Again she displayed no evidence of significant cognitive impairment when seen at the age of 26-years. Indeed she was very much valued in the workplace for remembering numerical codes for almost the entire stock inventory. AII.3 has a healthy daughter (AIII.1), born after an uneventful pregnancy. Evaluation of AIII.1 at the age of 3 weeks revealed no phenotypic abnormality. No intellectual problems were apparent in any other relatives of the three-generation family tree. DNA analysis of the FRAXA locus was normal and permission to use the remaining peripheral blood DNA for research purposes was obtained from patient AII.4 (Figure 1). An Epstein-Barr-transformed lymphoblastoid cell line was established at ECACC (Cambridge, UK) from a peripheral blood sample of proband AII.4. The index patient showed FRA2A expression in 40% of the examined cells. At the age of 20-years, chromosome analysis was repeated and confirmed FRA2A expression, this time in 21% of the examined cells. Subjects AII.3 and AIII.1 expressed the fragile site FRA2A in respectively 26% and 31% of the examined cells. The unaffected father (subject AI.1) showed FRA2A expression in 4% of his cells, whereas in two unaffected siblings and the mother no indications were found for FRA2A expression (Figure 1).

#### Family B

The proband (II.1, Figure 1) was born at 39 weeks after an uneventful pregnancy. He was the third child of a non-consanguineous Caucasian couple. There were no problems in the neonatal period. At 8 weeks of age, an anal fistula was diagnosed and required three surgical procedures between 5 and 8 months for cure. He developed mild asthma at 10 months of age. Food allergies were demonstrated at 12 months of age and were managed by dietary modification. His height and head circumference were within the normal range, but his development was slow. He had mild dysmorphic features, including telecanthus, slightly short palpebral fissures, smooth philtrum, thin upper lip and a small mouth. Psychological assessment at the age of 12 using the WISC-IV showed a full-scale IQ in the range 40–52 (<0.1 centile), within the moderate range of intellectual disability. The Vineland Adaptive Behaviour Scales 11 gave an Adaptive Behaviour Composite in the range 55–67, below the first centile and in the low range. His parents and two elder brothers had normal intelligence and were not dysmorphic. Routine chromosomes, subtelomere FISH of chromosomes, molecular testing for fragile X syndrome and urinary amino acids/organic acids/mucopolysaccharides gave normal results. The proband expressed the fragile site FRA2A in 40% of the examined cells. Fragile site expression was not examined in the other family members.

#### Family C

The proband (II.1, see Figure 1) was born at 33 weeks after an uneventful pregnancy. She had respiratory distress syndrome and was treated with surfactant and ventilated for five days. There were no significant complications in the neonatal period. Her early development was delayed and she suffered from intermittent asthma and required ventilator tubes for serious otitis media. At three years of age, she was seen because of speech delay – her expressive language was estimated to be at the 2 year level while receptive language and general development were assessed at the 2.5–3.0 year level. She was noted to be macrocephalic, hypotonic and hyperreflexic. Height was normal. At 13 years she is in an age appropriate class in high school and undertakes all subjects, except mathematics, with her peers, but struggles to keep up. She has poor concentration, is easily distractible and does not like changes in routine or to immediate expectations. She is doing well socially. The following investigations gave normal results: CT brain scan, urine amino acids and organic acids, chromosomes, creatine kinase and thyroid function tests. The proband showed FRA2A expression in 26% of the examined cells. Fragile site expression was not examined in the other family members.

### Fluorescent in situ hybridisation (FISH) mapping

Peripheral blood lymphocyte-derived metaphase chromosome preparations from individual AII.3 were obtained using standard methods. An *AFF3*-containing BAC-clone from the RPCI library, RP11-549H5 (AC092667), and clones mapping centromeric (RP11-436F6, AC010736) and telomeric (RP11-506F3, AC074387) to *AFF3* were obtained from the BACPAC Resource Center (Oakland, California, USA). Long Range-PCR (LR-PCR) was used to generate probes of 10 kb and 18 kb situated respectively immediately 5′ and 3′ to the promoter region of *AFF3*. The following primer pairs were used: L10K (forward 5′-TGCAGGAATGAATGAAGGGCAAGCAA-3′ and reverse 5′-TGGCCTCTGGGTGTCGACTTCAAACT-3′) and L18K (forward 5′-ACAGTTTGGCTTGACCGGGAGGGTTT-3′ and reverse 5′- TCAAAAATGTTCCCTTGCCCACAGTGC-3′). LR-PCR was performed using the Expand Long Template PCR System (Roche, Basel, Switzerland) according to the manufacturer's instructions. The amount of BAC DNA used per reaction was 5–10 ng. All probes were labelled with digoxigenin-11-dUTP or biotin-16-dUTP (Roche, Indianapolis, IN) by nick translation. DNA hybridisation and antibody detection were carried out as described previously [56]. At least five metaphases were analysed for each hybridisation, using a Zeiss Axioplan 2 fluorescence microscope equipped with a triple band-pass filter (#83000 for DAPI, FITC and Texas Red; Chroma Technology, Brattleboro, VT). Images were collected using a cooled CCD camera (Princeton Instruments Pentamax, Roper Scientific, Trenton, NJ) and analysed using IPLab software (Scanalytics, Vienna, VA).

### PCR amplification and hybridization of the FRA2A-associated CGG repeat

PCR amplification of the normal FRA2A CGG repeat was performed with the aid of 2.5× PCR Enhancer solution (Invitrogen, Carlsbad, CA, USA) using a forward primer P1 (5′-GGCCGTAAAAGCCACGAGAGAGGG-3′) and a reverse primer P2 (5′-CTTGCGCGCAGGCACACTCAAGAG-3′) derived from the sequences flanking the repeat. PCR products were sequenced and subsequently analysed by use of an ABI Prism 3130 Genetic Analyzer (Applied Biosystems, Foster City, CA, USA).

A Southern blot was created by digesting 10 µg of DNA, extracted from blood or Epstein-Barr-virus transformed cells, with the restriction enzymes *HindIII* and *NcoI* (Fermentas GmbH, St. Leon-Rot, Germany) in separate reactions. The digested DNA was then separated by electrophoresis on a 0.7% agarose gel. No ethidium bromide was added during this electrophoresis step to avoid product-related smearing on the gel that would cause overestimation of the mosaicism of repeat sizes [57]. After subsequent denaturation and neutralisation the DNA fragments were transferred to Hybond N^+^ membranes. Hybridisation was performed at 65°C using a specific 32P-labeled 992 bp PCR probe (forward primer 5′- AGCCTTTGTTCCTGGGAATGCTGTCTCAAT -3′ and reverse primer 5′- GGAAAGGCAGGTGATCAGCTAGAAGGGTG -3′).

Repeat primed PCR was performed to interrogate the number of CGG repeats in the *AFF3* gene with Asuragens CGG Repeat Primed *PCR* system designed for detection of Fragile X expanded alleles. Triplet repeat primed PCR (TP-PCR) uses a locus-specific forward primer that flanks the repeat. The reverse PCR primer of the primer pair is designed to hybridise within the CGG repeat region as it contains a (GCC)_5_ tail. This generates amplicons of various sizes as the reverse primers bind to multiple locations during TP-PCR. As the number of CGG repeats increases, a characteristic ladder profile appears on the fluorescence electropherogram enabling the rapid and inexpensive identification of expanded repeats that may have been missed using current PCR methods. Samples were PCR-amplified by preparing a master mix containing 11.45 µl of GC-rich AMP buffer, 0.25 µl of FAM-labelled *AFF3* forward primer (5′-GGCCGTAAAAGCCACGAGAGAGGG-3′), 0.25 µl of *AFF3* reverse primer (5′-CTTGCGCGCAGGCACACTCAAGAG-3′), 0.5 µl of CGG primer (5′- TACGCATCCCAGTTTGAGACGGCCGCCGCCGCCGCC-3′), 0.5 µl of nuclease-free water, and 0.05 µl of GC-rich polymerase mix from Asuragen (Austin, TX). 2 µl of the DNA sample, typically at 20 ng/µl, was added before transferring the plate to a thermal cycler (9700, Applied Biosystems, Foster City, CA). Samples were amplified with an initial heat denaturation step of 95°C for 5 minutes, followed by 10 cycles of 97°C for 35 seconds, 62°C for 35 seconds, and 68°C for 4 minutes, and then 20 cycles of 97°C for 35 seconds, 62°C for 35 seconds, and 68°C for 4 minutes with a 20 second autoextension at each cycle. The final extension step was 72°C for 10 minutes. After PCR, 2 µl of the PCR product was added to a mix with 11 µl HDF and 2 µl Rox 1000 standard. After a brief denaturation step, samples were analysed using the ABI Prism 3130 Genetic Analyzer.

### Methylation analysis of the *AFF3* promoter

The methylation status of the *AFF3* associated CGG repeat and the surrounding region, was analysed by bisulfite sequencing. Genomic DNA collected from lymphoblastoid cell lines and saliva (subject AIII.1) was bisulfite-treated using the EpiTect Bisulfite Kit (Qiagen, Venlo, Netherlands) according to manufacturer's guidelines. Bisulfite treatment converts all non-methylated cytosines into thymines, while methylated cytosines remain unchanged. Primers specific for the methylated bisulfite converted DNA (forward 5′- GGTGAGAAATAAAAAGAAAGGAG -3′ and reverse 5′- CCTCAACAACCCTAAAATACC -3′) were designed. After PCR amplification, the CGG surrounding area (chr2:100721494–100721911; hg19) was sequenced using an ABI Prism 3130 DNA sequencer. Moreover we have performed pyrosequencing using the AFF3_002 PyroMark CpG assay according to manufacturer's instructions (Qiagen, Venlo, Netherlands) and analysed the results on a PyroMark Q24. Methylation cut-off value was set at 10%.

### Gene-expression analysis

The expression pattern of *AFF3* in human tissues was studied using a multiple-tissue Northern blot (FirstChoice Northern Human Blot I, Ambion, Austin, TX, USA). The specific *AFF3* probe was a 655-bp PCR product [forward primer 5′-TATCGAGTGTGGAAATGCAA-3′ and reverse primer 5′-TGAGGTCCCTATGACAGGTG-3′] and radiolabelled by the addition of 32P-dATP and 32P-dCTP (MP, Irvine, California, USA). Hybridisation was performed according to the manufacturer's instructions.

Total RNA-sequencing data from the Illumina Human Body Map 2.0 project (GSE30611) was obtained from the NCBI Gene Expression Omnibus. Data from brain (ERR030882, female, 77y), ovary (ERR030874, female, 47yj) and lymph node (ERR030878, female, 86y) was downloaded in the form of 2×50 bp reads and imported into CLC genomics workbench v6.01. Transcriptomics analysis was performed within CLC Genomics using the human reference genome version hg19. Default settings were used, apart from a smaller expected insert size of 50 bp. Additionally total RNA was isolated from the human fetal brain tissues (FB1 54 gestational days (GD), FB2 47 GD, FB3 59 GD) according to the Trizol (Invitrogen) protocol. The preparation of amplicon libraries and RNA-Seq analysis were performed following standard Illumina TruSeq protocols and reads of length 50 bp were produced on the Illumina GAIIx platform. The fetal tissue was obtained following surgical termination of pregnancy and staged according to the Carnegie Classification [54], [55]. CAGE tag data was obtained from the FANTOM4 consortium as both pre-defined CAGE tag clusters (http://fantom.gsc.riken.jp/download/Tables/human/CAGE/promoters/tag_clusters/ and http://fantom.gsc.riken.jp/4/download/Tables/mouse/CAGE/promoters/tag_clusters/) and as genome aligned individual tags (http://fantom.gsc.riken.jp/4/download//Tables/human/CAGE/mapping/). Coordinates were converted from the hg18 reference genome assembly to hg19 using LiftOver (http://genome.ucsc.edu/cgi-bin/hgLiftOver). Statistical analysis was performed R (http://www.R-project.org/; version 3.0.0).

The *AFF3* transcript NM_002285 (http://www.ncbi.nlm.nih.gov/) was searched for cSNP's. The cSNP's in FRA2A patients were tested by PCR followed by sequencing at the genomic and at the cDNA level. RNA was isolated from Epstein-Barr-transformed lymphoblastoid cells using Trizol (Invitrogen, Carlsbad, CA, USA) and converted to cDNA using Superscript III reverse transcriptase (Invitrogen, Carlsbad, CA, USA). The following primer sets were used: SNP1 (rs4851214) in exon 14 (forward primer 5′- AGTGATGAAGAGGAGAATGAACA -3′ and reverse primer 5′- ATAGGAGGCTTGTGGGGATTA -3′) and SNP2 (rs13427251) in exon 25 (forward primer 5′-GTGTGTCTGGTATGTTTACAC-3′ and reverse primer 5′-GGATCAGCATCTAGTCTAAG-3′). Sequencing products were analysed on an ABI 3130 Prism automatic sequencer.

The *Aff3* riboprobe for whole mount in situ hybridisation to mouse embryos was generated by *in vitro* transcription from a PCR template amplified from the *Aff3* 3′UTR using mouse genomic DNA as a template. T3 (for sense probe) and T7 (for antisense probe) binding sites were added to the forward (5′-*AATTAACCCTCACTAAAGG*CTCTCCAACCGGATCCAGAAT-3′) and reverse (5′-*TAATACGACTCACTATAGG*AGCCCATGGCACCTCTCT-3′) primers. The WISH protocol and OPT scanning was performed exactly as previously described [58].

### Mutation analysis of the *AFF3* gene and marker analysis

All coding exons of the *AFF3* gene were PCR amplified at the genomic level using standard protocols for all patients and relatives to exclude the presence of any other disease-causing mutation. PCR products were enzymatically purified and sequenced. Sequences were analysed with an ABI Prism 3130 DNA sequencer.

For the marker analysis, genomic DNA was isolated from peripheral blood from all available family members using standard procedures. Highly polymorphic microsatellite markers, D2S2209 and D2S2311, were selected from the Marshfield genetic map in the proximity of the repeat. These markers are both dinucleotides with an average heterozygosity of 71%. Analysis was performed by a Go Taq DNA polymerase mediated PCR, with fluorescently labeled primers. Fragment analysis of amplified products was performed using an ABI PRISM 3130 XL Genetic Analyzer (Applied Biosystems). Allele identification was done with Gene mapper v3.7 software (Applied Biosystems).

## Supporting Information

Figure S1 CGG-repeat region in AFF3 intron 2. Sequence of the CGG-repeat region in intron 2 of the *AFF3* gene shown in telomeric-centrometic orientation. The CGG repeat is shown in bold blue text. The repeat lies within the 134 bp region that is deleted in subject AII.4 which is shown in bold black text. Forward and reverse primers used for the amplification of the CGG repeat are indicated with blue arrows. The primers used for bisulfite sequencing (chr2:100721494–100721911; hg19) are indicated with orange arrows and CpG sites that were analysed with bisulfite pyrosequencing are represented in bold orange text.
(TIF)

Figure S2 A: Fragment-length analysis of regular PCR and TP-PCR generated products of the CGG repeat in the *AFF3* gene of family A. Fluorescently-labeled PCR products of all individuals of family A were separated by capillary electrophoresis on an ABI PRISM 3130 XL Genetic Analyzer. For every individual a PCR covering the entire repeat was analyzed in addition to a repeat primed PCR (Asuragen). Individual AI.1 appeared homozygous for a repeat with 8 units as no fading repeat-signal is present after repeat-primed PCR (right corner). For individual AII.4 the 134 bp-deletion of the repeat and surrounding region is clearly detected in addition to a short 8-unit repeat. An expanded allele of over 300 units is present in this individual as shown with repeat primed PCR. This expanded allele could not be covered by regular PCR covering the entire repeat. In individuals AII.3 and AIII.1 a normal range repeat of 8 and 18 repeated units respectively was detected in addition to an expanded allele containing over 300 units. B: Fragment-length analysis of regular PCR and TP-PCR generated products of the CGG repeat in the *AFF3* gene of family B. In individual BI.2 one normal range allele with 15 repeated units was identified. In addition, a second slightly expanded allele of about 120 repeated units was detected by regular PCR covering the repeat. This expansion was confirmed with repeat primed PCR. In individual BII.1 a normal range repeat of 8 was detected in addition to an expanded allele containing over 300 units. The trace labelled FR_blanco represents a blanc reference lane. C: Fragment-length analysis of regular PCR and TP-PCR generated products of the CGG repeat in the *AFF3* gene of family C. The father of family C, CI.1, is heterozygous for the number of repeated units, displaying two alleles with respectively 5 and 8 repeated units. In the mother, CI.2, a second slightly expanded allele with 106 repeated units was detected in addition to a normal range allele with 17 CGG-units by regular PCR. This expansion was confirmed with repeat primed PCR. In individual CII.1 a normal range repeat of 8 was detected in addition to an expanded allele containing over 300 units. For each individual of this family the raw analysis data of the genetic analyzer are displayed in the upper right corner. The trace labelled FR_blanco represents a blanc reference lane.
(TIF)

Figure S3 Genotyping results of the microsatellite marker analysis on family A with markers D2S2209 and DS2311. From the combination of both markers we can conclude that both sisters (AII.3 and AII.4) have inherited a different allele from their father (AI.1), while they share a common allele that is probably inherited from the mother. It is again this allele that was passed on to the granddaughter (AIII.1). As individuals AII.3, AII.4 and AIII.1 all carry an expanded and hypermethylated allele for the *AFF3* associated repeat, it can be presumed that the expansion was probably inherited from the mother.
(TIF)

Figure S4 a: Bisulfite sequences of family A. After bisulfite treatment of the genomic DNA from lymphoblastoid cell lines and saliva (subject AIII.1) of all available family members, DNA sequences of the region located at chr2:100721494–100721911; hg19 were analysed with an ABI Prism 3130 DNA sequencer. This region covers 50 separate CpG dinucleotides of which nine are shown in this figure. Methylation patterns were consistent across all 50 CpG sites for each individual. Blue (C-) peaks at CpG-sites in addition to a red T-signal indicate the presence of at least partial methylation. b: Bisulfite sequences of family B. c: Bisulfite sequences of family C.
(TIF)

## References

[1] Wojciechowska M, Krzyzosiak WJ (2011) CAG repeat RNA as an auxiliary toxic agent in polyglutamine disorders. RNA Biol 8: 565–571. 21593608 10.4161/rna.8.4.15397 PMC3225975

[2] Costa Lima MA, Pimentel MM (2004) Dynamic mutation and human disorders: the spinocerebellar ataxias. Int J Mol Med 13: 3. 14719138

[3] Rudnicki DD, Margolis RL, Pearson CE, Krzyzosiak WJ (2012) Diced triplets expose neurons to RISC. Plos Genet 8: e1002545. 22383898 10.1371/journal.pgen.1002545 PMC3285583

[4] Pearson CE, Nichol Edamura K, Cleary JD (2005) Repeat instability: mechanisms of dynamic mutations. Nat Rev Genet 6: 729–742. 16205713 10.1038/nrg1689

[5] Kumari D, Usdin K (2009) Chromatin remodeling in the noncoding repeat expansion diseases. J Biol Chem 284: 5. 10.1074/jbc.R800026200 PMC2658035 18957431

[6] McMurray C (2010) Mechanisms of trinucleotide repeat instability during human development. Nat Rev Genet 11: 786–799. 20953213 10.1038/nrg2828 PMC3175376

[7] Dudek RW (2006) High-yield cell and molecular biology. Philadelphia; Baltimore; New York: Lippincott Williams & Wilkins.

[8] Licatalosi DD, Darnell RB (2006) Splicing regulation in neurologic disease. Neuron 52: 93–101. 17015229 10.1016/j.neuron.2006.09.017

[9] Pieretti M, Zhang FP, Fu YH, Warren ST, Oostra BA, et al (1991) Absence of expression of the FMR-1 gene in fragile X syndrome. Cell 66: 817–822. 1878973 10.1016/0092-8674(91)90125-i

[10] Trottier Y, Lutz Y, Stevanin G, Imbert G, Devys D, et al (1996) Polyglutamine expansion as a pathological epitope in Huntington's disease and four dominant cerebellar ataxias. Nature 378: 403–406. 7477379 10.1038/378403a0

[11] Lin YaW, JH (2011) Transcription-induced DNA toxicity at trinucleotide repeats. Cell Cycle 10: 611–618. 21293182 10.4161/cc.10.4.14729 PMC3173998

[12] Richards RI (2001) Fragile and unstable chromosomes in cancer: causes and consequences. Trends Genet 17: 339–345. 11377796 10.1016/s0168-9525(01)02303-4

[13] Richards RI (2001) Dynamic mutations: a decade of unstable expanded repeats in human genetic disease. Hum Mol Genet 10: 2187–2194. 11673400 10.1093/hmg/10.20.2187

[14] Zu T, Gibbens B, Doty NS, Gomes-Pereira M, Huguet A, et al (2011) Non-ATG-initiated translation directed by microsatellite expansions. Proc Natl Acad Sci U S A 108: 260–265. 21173221 10.1073/pnas.1013343108 PMC3017129

[15] Pearson CE (2011) Repeat associated non-ATG translation initiation: one DNA, two transcripts, seven reading frames, potentially nine toxic entities!. PLoS Genet 7: e1002018. 21423665 10.1371/journal.pgen.1002018 PMC3053344

[16] Benítez J (1999) Clinical and genetic implications of dynamic mutations in neuropediatric practice. Rev Neurol 28: 4. 10101767

[17] La Spada AR, Taylor JP (2010) Repeat expansion disease: progress and puzzles in disease pathogenesis. Nat Rev Genet 11: 13. 10.1038/nrg2748 PMC4704680 20177426

[18] Kato M (2006) A new paradigm for West syndrome based on molecular and cell biology. Epilepsy Res 70 Suppl 1: S87–95. 16806828 10.1016/j.eplepsyres.2006.02.008

[19] Debacker K, Kooy RF (2007) Fragile sites and human disease. Hum Mol Genet 16: R150–158. 17567780 10.1093/hmg/ddm136

[20] Verkerk AJMH, Pieretti M, Sutcliffe JS, Fu Y-H, Kuhl DPA, et al (1991) Identification of a gene (FMR-1) containing a CGG repeat coincident with a breakpoint cluster region exhibiting length variation in fragile X syndrome. Cell 65: 905–914. 1710175 10.1016/0092-8674(91)90397-h

[21] Knight SJL, Flannery AV, Hirst MC, Campbell L, Christodoulou Z, et al (1993) Trinucleotide repeat amplification and hypermethylation of a CpG island in *FRAXE* mental retardation. Cell 74: 127–134. 8334699 10.1016/0092-8674(93)90300-f

[22] Parrish JE, Oostra BA, Verkerk AJMH, Richards CS, Reynolds J, et al (1994) Isolation of a GCC repeat showing expansion in FRAXF, a fragile site distal to FRAXA and FRAXE. Nat Genet 8: 229–235. 7874164 10.1038/ng1194-229

[23] Nancarrow JK, Kremer E, Holman K, Eyre H, Doggett NA, et al (1994) Implications of FRA16A structure for the mechanism of chromosomal fragile site genesis. Science 264: 1938–1941. 8009225 10.1126/science.8009225

[24] Jones C, Penny L, Mattina T, Yu S, Baker E, et al (1995) Association of a chromosome deletion syndrome with a fragile site within the proto-oncogene *CBL2* . Nature 376: 145–149. 7603564 10.1038/376145a0

[25] Sarafidou T, Kahl C, Martinez-Garay I, Mangelsdorf M, Gesk S, et al (2004) Folate-sensitive fragile site FRA10A is due to an expansion of a CGG repeat in a novel gene, FRA10AC1, encoding a nuclear protein. Genomics 84: 69–81. 15203205 10.1016/j.ygeno.2003.12.017

[26] Winnepenninckx B, Debacker K, Ramsay J, Smeets D, Smits A, et al (2007) CGG repeat expansion in the *DIP2B* gene is associated with the fragile site FRA12A on chromosome 12q13.1. Am J Hum Genet 80: 221–231. 17236128 10.1086/510800 PMC1785358

[27] Debacker K, Winnepenninckx B, Longman C, Colgan J, Tolmie J, et al (2007) The molecular basis of the folate-sensitive fragile site FRA11A at 11q13. Cytogenet Genome Res 119: 9–14. 18160775 10.1159/000109612

[28] Pieretti M, Zhang F, Fu Y-H, Warren ST, Oostra BA, et al (1991) Absence of expression of the FMR-1 gene in fragile X syndrome. Cell 66: 817–822. 1878973 10.1016/0092-8674(91)90125-i

[29] Kooy RF (2009) Fragile sites and human disease. Encyclopedia of Life Sciences. Chichester, UK: John Wiley & Sons, Ltd. pp. DOI:10.1002/9780470015902.a9780470021457.

[30] Tukun A, Renda Y, Topcu M, Tuncali T, Bokesoy I (2000) Mental retardation with rare fragile site expressed at 2q11. Brain Dev 22: 498–500. 11111064 10.1016/s0387-7604(00)00189-3

[31] Murthy DS, Teebi AS, Sundareshan TS, al-Awadi SA (1990) Familial fragile secondary constriction on chromosome 2 (2q11) with unusual features and psychomotor retadation. Indian Pediatr 57: 257–260. 10.1007/BF02722098 2246023

[32] Annerén G, Gustavson KH (1981) A fragile secondary constriction on chromosome 2 in five patients with different clinical features. Hereditas 95: 63–67. 7333874 10.1111/j.1601-5223.1981.tb01329.x

[33] Ma C, Staudt L (1996) LAF-4 encodes a lymphoid nuclear protein with transactivation potential that is homologous to AF-4, the gene fused to MLL in t(4;11) leukemias. Blood 87: 734–745. 8555498

[34] Kodzius R, Kojima M, Nishiyori H, Nakamura M, Fukuda S, et al (2006) CAGE: cap analysis of gene expression. Nat Methods 3: 211–222. 16489339 10.1038/nmeth0306-211

[35] Tost J, Gut IG (2007) DNA methylation analysis by pyrosequencing. Nat Protocols 2: 2265–2275. 17853883 10.1038/nprot.2007.314

[36] Illingworth R, Kerr A, Desousa D, Jorgensen H, Ellis P, et al (2008) A novel CpG island set identifies tissue-specific methylation at developmental gene loci. PLoS Biol 6: e22. 18232738 10.1371/journal.pbio.0060022 PMC2214817

[37] Lancaster MA, Renner M, Martin CA, Wenzel D, Bicknell LS, et al (2013) Cerebral organoids model human brain development and microcephaly. Nature 501: 373–379. 23995685 10.1038/nature12517 PMC3817409

[38] de Graaff E, Rouillard P, Willems PJ, Smits APT, Rousseau F, et al (1995) Hotspot for deletions in the CGG repeat region of *FMR1* in fragile X patients. Hum Mol Genet 4: 45–49. 7711733 10.1093/hmg/4.1.45

[39] Axford MM, Lopez-Castel A, Nakamori M, Thornton CA, Pearson CE (2011) Replacement of the myotonic dystrophy type 1 CTG repeat with ‘non-CTG repeat’ insertions in specific tissues. J Med Genet 48: 438–443. 21622935 10.1136/jmg.2010.085944 PMC3379714

[40] Gronskov K, Hjalgrim H, Bjerager MO, Brondum-Nielsen K (1997) Deletion of all CGG repeats plus flanking sequences in FMR1 does not abolish gene expression. Am J Hum Genet 61: 961–967. 9382110 10.1086/514872 PMC1716002

[41] Taki T, Kano H, Taniwaki M, Sako M, Yanagisawa M, et al (1999) *AF5q31*, a newly identified *AF4*-related gene, is fused to *MLL* in infant acute lymphoblastic leukemia with ins(5;11) (q31;q13q23). Proc Natl Acad Sci USA 96: 14535–14540. 10588740 10.1073/pnas.96.25.14535 PMC24471

[42] Liao X, Ma C, Trask B, Massa H, Gilbert D, et al (1996) LAF4 maps to mouse chromosome 1 and human chromosome 2q11.2-q12. Mamm Genome 7: 467–468. 8662235 10.1007/s003359900137

[43] Melko M, Douguet D, Bensaid M, Zongaro S, Verheggen C, et al (2011) Functional characterization of the AFF (AF4/FMR2) family of RNA-binding proteins: insights into the molecular pathology of FRAXE intellectual disability. Hum Mol Genet 20: 1873–1885. 21330300 10.1093/hmg/ddr069

[44] Britanova O, Lukyanov S, Gruss P, Tarabykin V (2002) The mouse Laf4 gene: Exon/intron organization, cDNA sequence, alternative splicing, and expression during central nervous system development. Genomics 80: 31–37. 12079280 10.1006/geno.2002.6796

[45] Gécz J, Oostra BA, Hockey A, Carbonell P, Turner G, et al (1997) FMR2 expression in families with *FRAXE* mental retardation. Hum Mol Genet 6: 435–441. 9147647 10.1093/hmg/6.3.435

[46] Chakrabarti L, Bristulf J, Foss GS, Davies KE (1998) Expression of the murine homologue of FMR2 in mouse brain and during development. Hum Mol Genet 7: 441–448. 9467002 10.1093/hmg/7.3.441

[47] Mondal K, Ramachandran D, Patel VC, Hagen KR, Bose P, et al (2012) Excess variants in AFF2 detected by massively parallel sequencing of males with autism spectrum disorder. Hum Mol Genet 21: 4356–4364. 22773736 10.1093/hmg/dds267 PMC3441129

[48] Gu Y, Nelson DL (2003) FMR2 function: insight from a mouse knockout model. Cytogenet Genome Res 100: 129–139. 14526173 10.1159/000072847

[49] Davies KE, Oliver PL, Jones EL, Jeans A, Clark J, et al (2003) Functional studies of Af4 in the robotic mouse. Am J Hum Genet 73 Suppl: A86.

[50] Steichen-Gersdorf E, Gassner I, Superti-Furga A, Ullmann R, Stricker S, et al (2008) Triangular tibia with fibular aplasia associated with a microdeletion on 2q11.2 encompassing LAF4. Clin Genet 74: 560–565. 18616733 10.1111/j.1399-0004.2008.01050.x

[51] Willemsen R, Bontekoe CJ, Severijnen LA, Oostra BA (2002) Timing of the absence of FMR1 expression in full mutation chorionic villi. Hum Genet 110: 601–605. 12107447 10.1007/s00439-002-0723-5

[52] Ruiz-Herrera A, Garcia F, Giulotto E, Attolini C, Egozcue J, et al (2005) Evolutionary breakpoints are co-localized with fragile sites and intrachromosomal telomeric sequences in primates. Cytogenet Genome Res 108: 234–247. 15545736 10.1159/000080822

[53] Ruiz-Herrera A, Castresana J, Robinson TJ (2006) Is mammalian chromosomal evolution driven by regions of genome fragility? Genome Biol 7: R115. 17156441 10.1186/gb-2006-7-12-r115 PMC1794428

[54] O'Rahilly R, Muller F (1987) Developmental stages in human embryos: revised and new measurements. Cells Tissues Organs 192: 73–84. 20185898 10.1159/000289817

[55] Bullen P, Wilson D, editors(1997) The Carnegie Staging of Human Embryos: a Practical Guide. Oxford: Bios Scientific Publishers. 27–50 p.

[56] Chong SS, Pack SD, Roschke AV, Tanigami A, Carrozzo R, et al (1997) A revision of the lissencephaly and Miller-Dieker syndrome critical regions in chromosome 17p13.3. Hum Mol Genet 6: 147–155. 9063734 10.1093/hmg/6.2.147

[57] Nolin SL, Brown WT, Glicksman A, Houck GE Jr, Gargano AD, et al (2003) Expansion of the fragile X CGG repeat in females with premutation or intermediate alleles. Am J Hum Genet 72: 454–464. 12529854 10.1086/367713 PMC379237

[58] Rainger J, Bengani H, Campbell L, Anderson E, Sokhi K, et al (2012) Miller (Genee-Wiedemann) syndrome represents a clinically and biochemically distinct subgroup of postaxial acrofacial dysostosis associated with partial deficiency of DHODH. Hum Mol Genet 21: 3969–3983. 22692683 10.1093/hmg/dds218

