# Supplementary figures and images for: FRA2A Is a CGG Repeat Expansion Associated with Silencing of AFF3
Source: PLoS Genet. 2014 Apr 24;10(4):e1004242. doi: 10.1371/journal.pgen.1004242 (PMC3998887; doi:10.1371/journal.pgen.1004242)

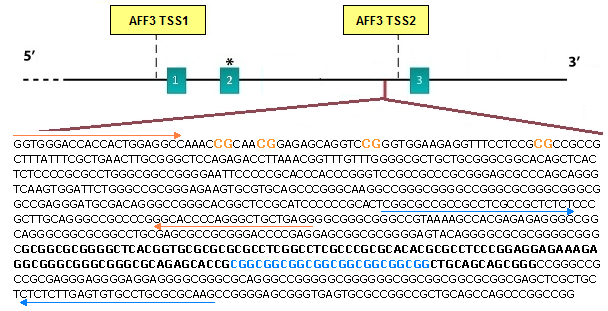

Supplement: Figure S1 — CGG-repeat region in AFF3 intron 2. Sequence of the CGG-repeat region in intron 2 of the AFF3 gene shown in telomeric-centrometic orientation. The CGG repeat is shown in bold blue text. The repeat lies within the 134 bp region that is deleted in subject AII.4 which is shown in bold black text. Forward and reverse primers used for the amplification of the CGG repeat are indicated with blue arrows. The primers used for bisulfite sequencing (chr2:100721494–100721911; hg19) are indicated with orange arrows and CpG sites that were analysed with bisulfite pyrosequencing are represented in bold orange text. (TIF) [file pgen.1004242.s001.tif]

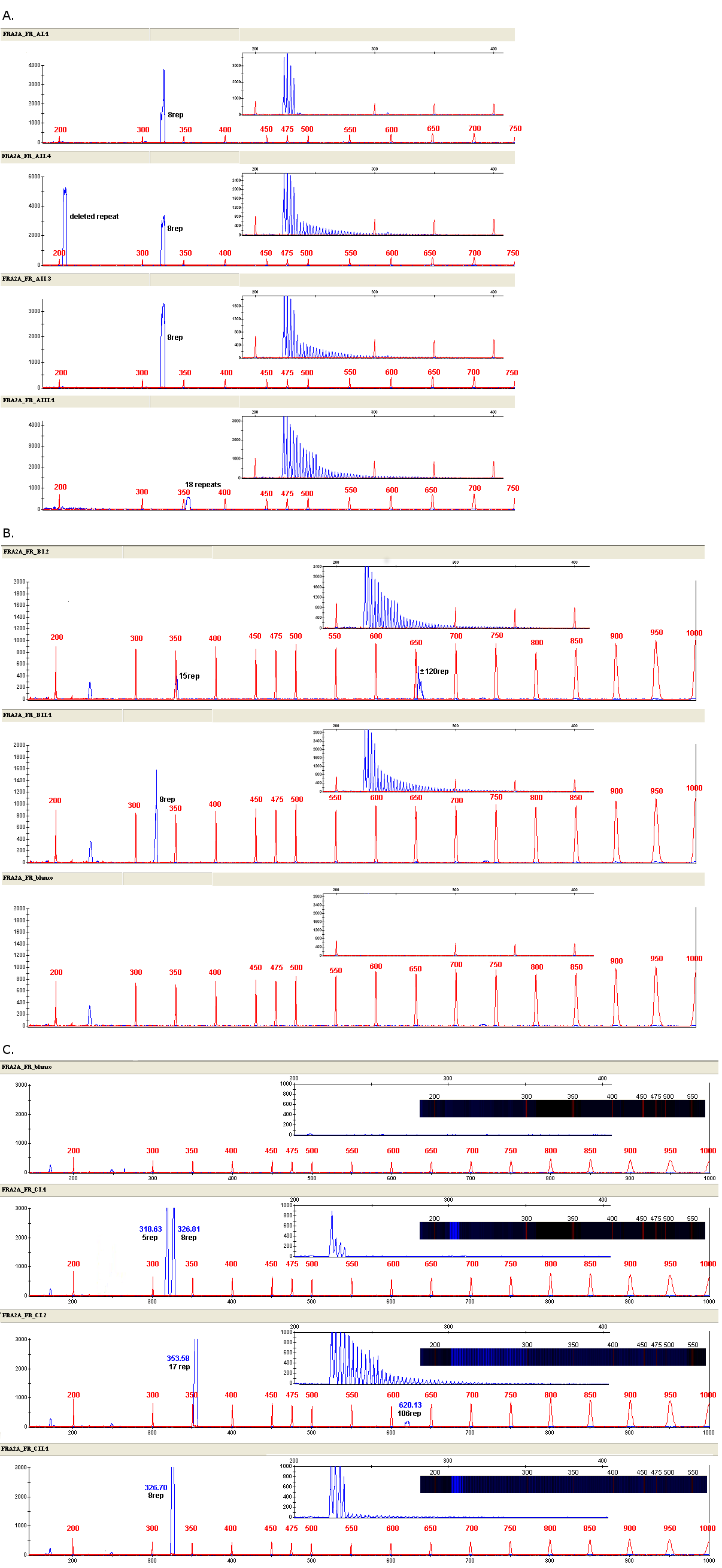

Supplement: Figure S2 — A: Fragment-length analysis of regular PCR and TP-PCR generated products of the CGG repeat in the AFF3 gene of family A. Fluorescently-labeled PCR products of all individuals of family A were separated by capillary electrophoresis on an ABI PRISM 3130 XL Genetic Analyzer. For every individual a PCR covering the entire repeat was analyzed in addition to a repeat primed PCR (Asuragen). Individual AI.1 appeared homozygous for a repeat with 8 units as no fading repeat-signal is present after repeat-primed PCR (right corner). For individual AII.4 the 134 bp-deletion of the repeat and surrounding region is clearly detected in addition to a short 8-unit repeat. An expanded allele of over 300 units is present in this individual as shown with repeat primed PCR. This expanded allele could not be covered by regular PCR covering the entire repeat. In individuals AII.3 and AIII.1 a normal range repeat of 8 and 18 repeated units respectively was detected in addition to an expanded allele containing over 300 units. B: Fragment-length analysis of regular PCR and TP-PCR generated products of the CGG repeat in the AFF3 gene of family B. In individual BI.2 one normal range allele with 15 repeated units was identified. In addition, a second slightly expanded allele of about 120 repeated units was detected by regular PCR covering the repeat. This expansion was confirmed with repeat primed PCR. In individual BII.1 a normal range repeat of 8 was detected in addition to an expanded allele containing over 300 units. The trace labelled FR_blanco represents a blanc reference lane. C: Fragment-length analysis of regular PCR and TP-PCR generated products of the CGG repeat in the AFF3 gene of family C. The father of family C, CI.1, is heterozygous for the number of repeated units, displaying two alleles with respectively 5 and 8 repeated units. In the mother, CI.2, a second slightly expanded allele with 106 repeated units was detected in addition to a normal range allele with 17 CGG-units by reg [file pgen.1004242.s002.tif]

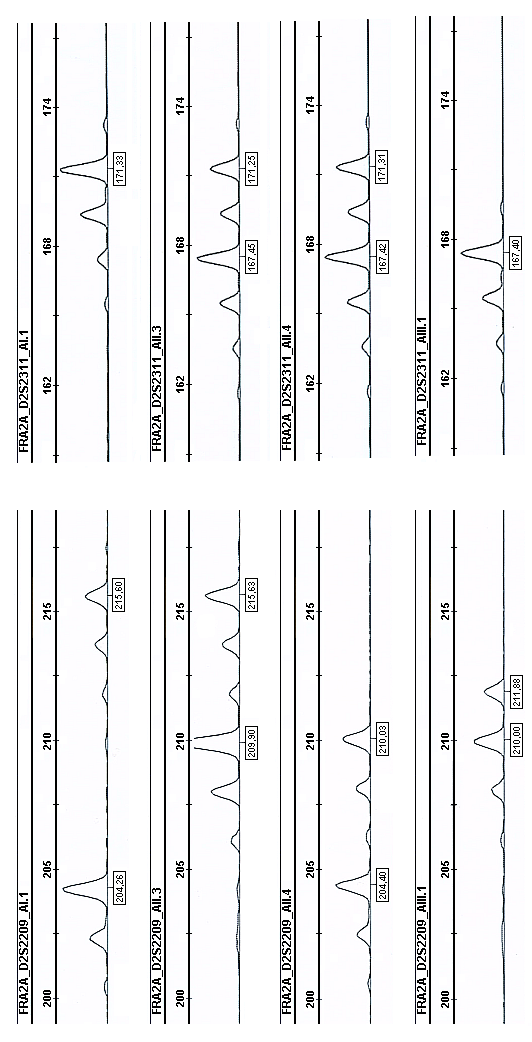

Supplement: Figure S3 — Genotyping results of the microsatellite marker analysis on family A with markers D2S2209 and DS2311. From the combination of both markers we can conclude that both sisters (AII.3 and AII.4) have inherited a different allele from their father (AI.1), while they share a common allele that is probably inherited from the mother. It is again this allele that was passed on to the granddaughter (AIII.1). As individuals AII.3, AII.4 and AIII.1 all carry an expanded and hypermethylated allele for the AFF3 associated repeat, it can be presumed that the expansion was probably inherited from the mother. (TIF) [file pgen.1004242.s003.tif]

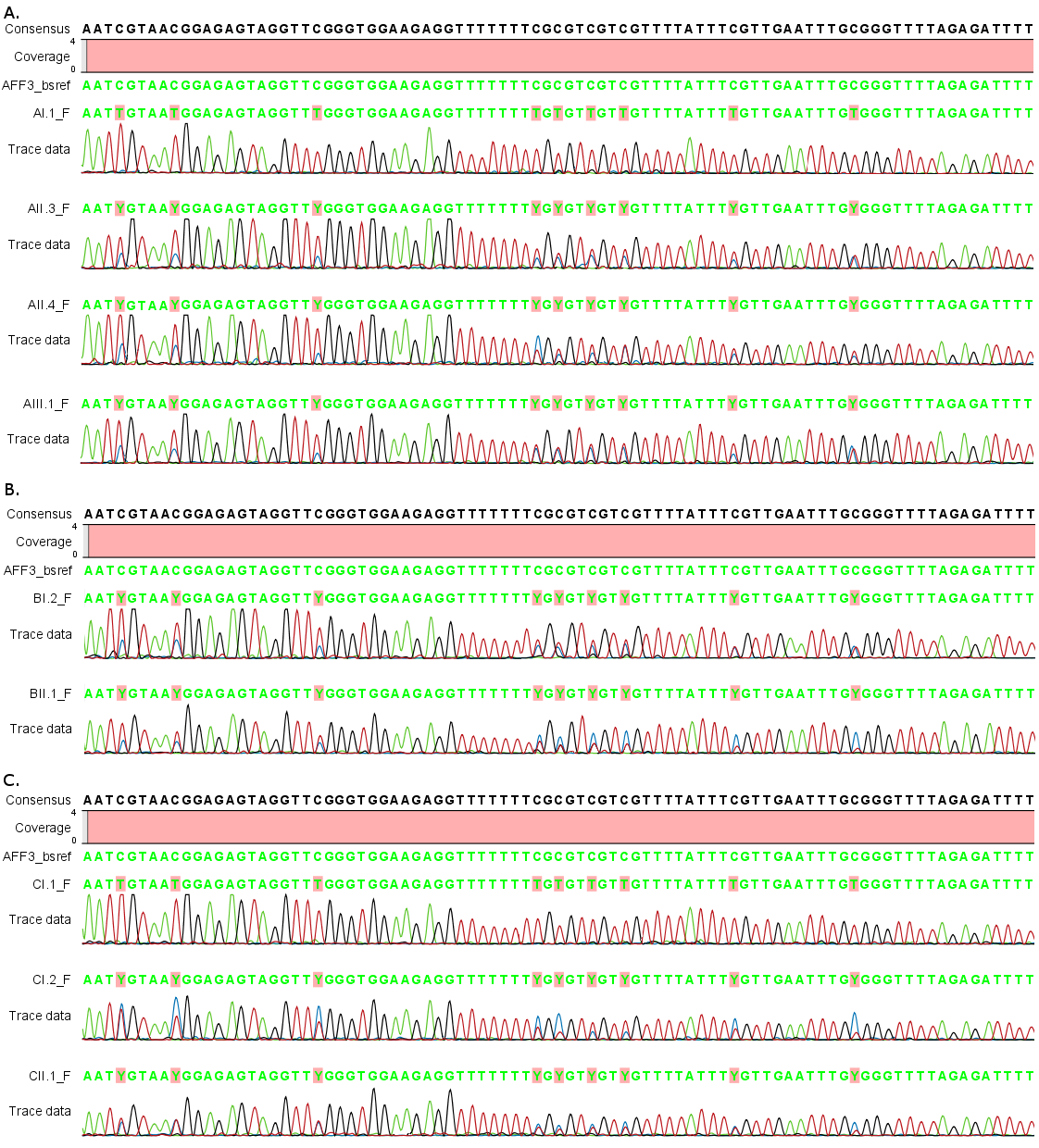

Supplement: Figure S4 — a: Bisulfite sequences of family A. After bisulfite treatment of the genomic DNA from lymphoblastoid cell lines and saliva (subject AIII.1) of all available family members, DNA sequences of the region located at chr2:100721494–100721911; hg19 were analysed with an ABI Prism 3130 DNA sequencer. This region covers 50 separate CpG dinucleotides of which nine are shown in this figure. Methylation patterns were consistent across all 50 CpG sites for each individual. Blue (C-) peaks at CpG-sites in addition to a red T-signal indicate the presence of at least partial methylation. b: Bisulfite sequences of family B. c: Bisulfite sequences of family C. (TIF) [file pgen.1004242.s004.tif]
